# Lugol’s Iodine as a Key Treatment in Transient Gestational Thyrotoxicosis: A Case Report

**DOI:** 10.1155/crie/7377427

**Published:** 2026-07-16

**Authors:** Mariam Mabrouk, Najla Bchir, Dorra El Guiche, Anaam Ben Chehida, Emna Mraihi, Chadia Zouaoui, Haroun Ouertani

**Affiliations:** ^1^ Department of Endocrinology, Military Hospital of Tunis, Tunis, Tunisia, hopmil.defense.tn

**Keywords:** case report, gestational thyrotoxicosis, hyperthyroidism, Lugol’s iodine, pregnancy

## Abstract

**Introduction:**

Transient gestational thyrotoxicosis (TGT) occurs when human chorionic gonadotropin (hCG) directly stimulates the maternal thyroid gland during the first trimester of pregnancy. We report a case of complicated TGT in a young woman who required treatment with Lugol’s solution.

**Case Presentation:**

A 29‐year‐old pregnant woman, at 10 weeks gestation and with no significant medical history, was referred to the endocrinology department for evaluation of hyperthyroidism. She had been experiencing persistent vomiting, anorexia, fatigue, palpitations, abdominal pain, and unintentional weight loss for 2 weeks. On examination, her heart rate was at 120 bpm, and she exhibited resting tremors, but there were no signs of exophthalmos or goiter. Laboratory results revealed a free T4 level 2.2 times above the upper limit of the normal range, with a thyroid‐stimulating hormone (TSH) level of 0.01 mUI/L. Tests for thyroid receptor antibodies, thyroid peroxidase antibodies, and anti‐thyroglobulin antibodies were negative. A cervical ultrasound with Doppler showed a normal‐sized thyroid gland without hypervascularity or nodules, confirming the diagnosis of TGT.

Additionally, liver enzyme abnormalities were observed, with ASAT elevated 4.84 times and ALAT 3.42 times the normal limit. Also, acute pancreatitis was diagnosed, as evidenced by a lipase level three times above the upper limit of normal. Given the contraindications for antithyroid drugs (ATDs) and glucocorticoids, treatment was initiated with Lugol’s iodine 5% (five drops three times daily) and propranolol (60 mg/day) for 2 weeks. Symptoms of thyrotoxicosis resolved, free T4 returned to normal levels, and liver enzymes normalized.

**Conclusion:**

This case highlights the interesting role of Lugol’s iodine as a short‐term and effective therapeutic option in selected cases of TGT.

## 1. Introduction

Peripheral hyperthyroidism refers to an increase in serum free thyroxine (T4) and triiodothyronine (T3) associated with a decrease in TSH levels [1]. During pregnancy, hyperthyroidism is most frequently either caused by Graves’ disease or transient gestational thyrotoxicosis (TGT), two entities that differ markedly in pathophysiology, prognosis, and management. TGT occurs in 2%–11% of all pregnancies, particularly during the first trimester. Due to its structural homology with TSH, human chorionic gonadotropin (hCG) can bind to and activate TSH receptors on thyroid follicular cells, leading to increased thyroid hormone synthesis and release. This stimulatory effect is usually transient and parallels the physiological rise and subsequent decline of hCG concentrations as pregnancy progresses. TGT is frequently associated with hyperemesis gravidarum and typically resolves spontaneously by mid‐gestation as hCG levels decrease [2].

Management of hyperthyroidism during pregnancy depends on the underlying etiology and the severity of clinical and biochemical manifestations. Graves’ disease generally requires treatment with antithyroid drugs (ATDs), most commonly propylthiouracil during the first trimester, followed by methimazole thereafter, while carefully balancing maternal benefits against potential fetal risks [3]. In contrast, TGT is usually characterized by mild, self‐limiting thyrotoxicosis and does not require ATDs. Supportive measures, including hydration, correction of electrolyte disturbances, and short‐term use of beta‐blockers for symptomatic relief, are typically sufficient [4].

In rare cases of severe TGT with significant clinical impact, standard supportive management may be insufficient, and the use of ATDs may be contraindicated or poorly tolerated. Alternative therapeutic strategies in such situations are not well established and are rarely reported.

Herein, we report the rare case of a 29‐year‐old woman with severe TGT who required treatment with lugol’s iodine. This case is noteworthy due to the rare use of Lugol’s iodine as a therapeutic option in TGT, highlighting an alternative approach when conventional ATDs are contraindicated or not tolerated. This case report follows the CARE Guidelines [5].

## 2. Case Presentation

A 29‐year‐old woman, G4P3, at 10 weeks gestation was referred to the endocrinology department for evaluation and management of hyperthyroidism. Her medical history included hyperemesis gravidarum in her previous pregnancies, for which she was hospitalized and received serum infusion and electrolyte intravenous supplementation. She has no personal or family history of autoimmune disease. On interrogation, she reported incoercible vomiting, anorexia, asthenia, palpitations, abdominal pain, and involuntary weight loss ongoing for a period of 2 weeks. Physical examination revealed a body mass index (BMI) of 22.67 Kg/m^2^, normal capillary blood glucose level. Her heart rate was at 120 bpm. Arterial blood pressure (BP) was at 100/60 mmHg. She had resting tremors and pale conjunctivae. No exophtalmia or goiter was noted. An electrocardiogram was performed, showing sinus tachycardia at 116 bpm, with no arrythmia or ST segment abnormalities.

Laboratory testing was performed. Nonregenerative normochromic normocyte anemia was noted. Fasting blood glucose was at 3.1 mmol/L. Total bilirubin levels were at 27 umol/L (normal <17 umol/L). Gamma glutamyl transferase (GGT) was at 76 mUI/L (normal range: 7–64). TGO and TGP levels elevated 3.26 and 4.83 times above the normal range, respectively. Additionally, acute pancreatitis was diagnosed, supported by abdominal pain and a lipase level at three times the upper limit of normal. Abdominal CT imaging was not performed because of pregnancy‐related contraindications. Other common causes of acute pancreatitis, including gallstone disease, alcohol consumption, hypertriglyceridemia, and drug‐induced pancreatitis, were systematically excluded. Free T4 was 2.2 times above the upper limit of the laboratory reference range for the first trimester of pregnancy (6.7–13.9 pmol/L), and the TSH level was 0.01 mIU/L (reference range: 0.1–2.5 mIU/L for the first trimester of pregnancy) (Table 1). Cervical sonography was performed and revealed a normal‐sized thyroid gland without hypervascularity or thyroid nodules. Also, testing for thyroid‐stimulating receptor antibodies (TRAb), thyroid peroxidase antibodies, and anti‐thyroglobulin antibodies was negative, supporting the diagnosis of TGT.

**Table 1:** Biochemical and hormonal profile at diagnosis.

Parameter	Value	Normal range
Hemoglobin (g/dL)	11	13–18
Hematocrit (%)	33.4	40–52
MCV (fL)	81.5	82–97
MCH (pg/c)	26.8	27–32
MCHC (%)	33	32–36
WBC (c/l)	4900	4000–10,000
ANC (c/l)	3400	1700–7000
Platelet count (c/l)	151,000	150,000–400,000
Urea (mmol/L)	2.2	3.3–7.0
Creatininemia	26	53–120
Fasting blood glucose (mmol/L)	3.1	4–5.9
Calcium level (mmol/L)	2.29	0.80–1.61
Sodium level (mmol/L)	138	136–145
Potassium level (mmol/L)	3.9	3.5–4.5
C reactive protein (mg/L)	<8	<8
Total bilirubin (umol/L)	27	<17
Lipase (UI/L)	553	<190
Alcaline phosphatase (UI/L)	71	42–121
Gamma GT (UI/L)	76	7–64
TGO (UI/L)	137 (3.26 × N)	10–42
TGP (UI/L)	290 (4.83 × N)	10–60
Free T4 (pmol/L)	30.7 (2.2 × N)	6.7–13.9
TSH (mUI/L)	0.01	0.1–2.5

Management included strict rest and comprehensive supportive care for hyperemesis gravidarum and dehydration. Intravenous fluid therapy with isotonic crystalloids was administered to correct dehydration, and electrolyte disturbances were closely monitored and corrected. Antiemetic therapy and nutritional support were also provided. Beta‐blocker therapy with propranolol (60 mg/day) was initiated to control symptoms of thyrotoxicosis. ATDs and glucocorticoids were contraindicated due to significant hepatic cytolysis. Therefore, the patient was started with Lugol’s iodine 5% at a dose of 5 drops (one drop = 8 mg iodide) three times daily.

**Figure 1:**
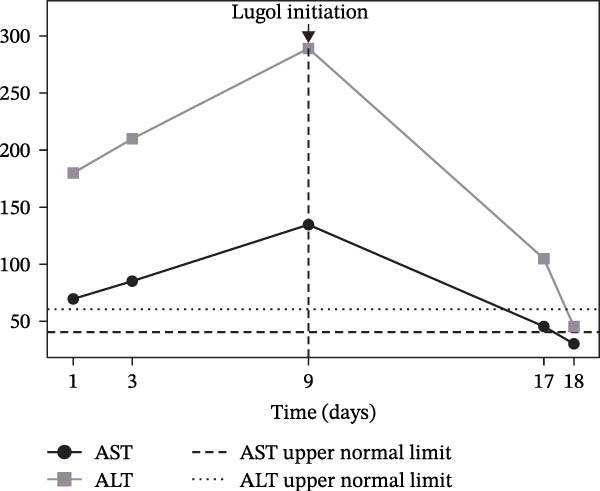
Evolution of liver enzyme serum levels under treatment.

After 2 weeks of treatment, there was a significant clinical improvement. Vomiting ceased, and the patient regained her appetite. Abdominal pain and palpitations were gone. Her heart rate decreased to 82 bpm and she no longer had tremors. Liver and pancreatic enzymes normalized (Figure 1). On Day 21, Free T4 was within the normal range, while TSH remained low at 0.05 mUI/L. On Day 28, both TSH and Free T4 levels had normalized (Table 2). The patient had an uncomplicated vaginal delivery; and the postpartum period was uneventful for both the mother and the neonate.

**Table 2:** Evolution of thyroid hormone levels under treatment.

Parameter	Before Lugol	Under Lugol treatment	
	Day 1	Day 21	Day 28
Free T4 (pmol/L)	30.7	7.8	8.5
TSH (mUI/L)	0.01	0.05	2.12

## 3. Discussion

Nausea and vomiting affect 50% to 90% of women during the first trimester of pregnancy [6] and it is crucial for clinicians to consider and rule out hyperthyroidism in all women presenting with these symptoms. When hyperthyroidism is diagnosed, two main causes ought to be distinguished: Graves’ disease and TGT. TGT is due to very high concentrations of hCG, a hormone produced by the trophoblast [7]. hCG is structurally homologous to thyroid‐stimulating hormone (TSH) [2]. Therefore, it stimulates TSH receptors, resulting in high levels of free T4 during the first trimester of pregnancy [2, 6]. Sun et al. [8] evaluated the frequency of TGT in 143 women with hyperemesis gravidarum. The incidence of TGT was significantly higher when hCG levels exceeded 80,000 UI/L and clinical TGT was present when hCG levels were above 180,000 UI/L. Moreover, serum hCG correlated negatively with TSH levels and positively with free T4 levels [8, 9]. In the case presented, serum hCG was not measured, which represents a limitation. However, it is not part of the formal diagnostic criteria for TGT, and the diagnosis was established based on the clinical presentation, thyroid function tests, exclusion of autoimmune hyperthyroidism, and the resolving course [3]. Prevalence of TGT varies from 2% to 11%, which is 10‐folds more frequent than Graves’ disease [1, 8, 10] and is often recurrent in pregnancies [6]. Our patient had a history of hyperemesis gravidarum during her previous pregnancies. Hyperemesis started during the 10th week of pregnancy in our patient. This can be explained by the kinetics of hCG, reaching 50,000–100,000 UI/L in the 10th week of gestation, which is accompanied by a fall in TSH serum levels [11]. She had no symptoms or signs of hyperthyroidism before pregnancy. On physical examination, hyperthyroidism was clinically mild, manifesting in tachycardia and resting tremor. This further supported the diagnosis of TGT in our patient, as it is typically associated with mild or absent thyrotoxic symptoms. Graves’ disease was considered in the differential diagnosis but was deemed unlikely based on clinical, biological, and imaging findings. The absence of goiter, ophthalmopathy, and thyroid hypervascularity argued against an autoimmune etiology. In addition, there was no relevant personal or family history suggestive of autoimmune thyroid disease. The rapidly favorable clinical course, negative TRAb, and follow‐up findings further supported the exclusion of Graves’ disease, in favor of a diagnosis of TGT [12]. Glinoer [10] screened 1900 pregnant women for the presence of biological hyperthyroidism. TGT was present in 18 cases, and from a clinical point of view, symptoms such as weight loss, the absence of weight gain, unexplained fatigue and tachycardia were present in only half of them. Hyperemesis was associated with the most severe cases that required hospitalization for 1–2 weeks. In another study, 65 of 143 patients with hyperemesis gravidarum were diagnosed with TGT, and none of them manifested obvious signs of hyperthyroidism [8].

Antithyroid antibodies were negative for our patient, confirming the diagnosis of TGT. However, free T4 levels were 2.2 times above the normal range, while TSH levels were at 0.01 UI/L. An other study [6] reported the case of a 20‐year‐old woman with TGT who had significantly low TSH serum levels, under 0.02, and free T4 at 1.8 times above the normal range. It is important to first discuss the accuracy of free T4 laboratory measurement in the diagnosis of hyperthyroidism during pregnancy. Indeed, free T3 and T4 levels increase by 50% higher than the nonpregnant concentrations and remain high throughout the pregnancy [2]. This can be explained by the increase in thyroid‐binding globulin (TBG) from Weeks 7 to 20 of gestation [2, 13, 14] which may increase free T4 levels by 50% from week 7 to week 16 of gestation [4]. Furthermore, there is a decrease in albumin levels during pregnancy [14] which may influence the interpretation of the hormonal workup. While the American Thyroid Association (ATA) recommends the use of a pregnancy‐adjusted reference range for the diagnosis of hyperthyroidism during pregnancy [4].

Our patient had high TGO and TPO serum levels and bilirubin levels, in contrast to mild clinical and biological hyperthyroidism. This can be explained by the severity of emesis. In fact, hepatic cytolysis was proven to be correlated to the importance of vomiting [15]. It can also be explained by hyperthyroidism. Elevated liver blood tests are a common complication of thyrotoxicosis, with a frequency of 60%–67% regardless of the etiology [16]. Pornchai et al. [17] reported the case of a 22‐year‐old pregnant woman at 9 weeks of gestation, diagnosed with TGT and presented with jaundice. On biological examination, she had elevated liver enzymes: ALAT and ASAT were 10 and 12 times above the normal range, respectively. Bilirubin level was 10.8 times the upper normal limit. Other causes of hepatic cytolysis were excluded. After supportive care including vitamin therapy, intravenous hydration and electrolyte supplementation, liver enzymes decreased after 4 days of her hospital admission and by week 10 of gestation, jaundice subsided. These findings conclude to a potential role of hyperemesis gravidarum in the onset of severe hepatic cytolysis, reversible after symptomatic treatment.

Although rare, an association between thyrotoxicosis and acute pancreatitis has been reported. A recent case report documented acute pancreatitis occurring simultaneously with thyrotoxicosis, underscoring that thyrotoxicosis may contribute to pancreatic inflammation even in the absence of typical causes such as gallstones or alcohol use [18]. Experimental evidence also suggests that thyroid hormones can influence pancreatic acinar cell responses during pancreatitis, providing a potential mechanistic basis for interaction between thyroid function and pancreatic pathology [19]. The absence of other common etiologies and the parallel improvement of both conditions in our patient support a possible causal relationship.

Management of TGT during pregnancy is based on management of dehydration, hospitalization and beta‐blockers [4], which was the primary treatment for our patient. She had severe dehydration due to the long‐term vomiting, anorexia, and high free T4 levels. Therefore, antithyroid therapy was considered to alleviate the symptoms and improve her general state. However, these molecules were not recommended in TGT in general [4] and contraindicated in our patient’s case. ATDs were avoided because the patient was in the first trimester of pregnancy, a period during which methimazole is associated with an increased risk of congenital malformations, while propylthiouracil carries a recognized risk of hepatotoxicity [20]. Moreover, in TGT, which is mediated by hCG stimulation and typically self‐limiting, antithyroid therapy is not recommended [21]. Glucocorticoids were also not advised because of the hepatic cytolysis. The therapeutic choice was therefore guided by pregnancy‐related safety concerns and the underlying pathophysiology of the disorder. In this context, a short course of Lugol’s iodine was selected as a practical, time‐limited strategy to obtain rapid biochemical control while limiting potential maternal and fetal risks. Lugol’s iodine intake inhibits the enzyme thyroid peroxidase that is responsible for the oxidation and the organification of thyroid hormones [22]. Thyroid hormone synthesis is therefore blocked [23]. By all means, the efficacy of Lugol in normalizing thyroid hormone levels is undeniable. It was proven in one study that the control of hyperthyroidism in Graves’ disease after 12 months of iode solution was comparable to that seen in patients treated with low doses of methimazole [24]. One drawback of this treatment option is the temporary effect of thyroid suppression. Indeed, in a study by Takata et al. [25], methimazole was used together with iodide solution for 8 weeks, and the latter was discontinued when free T4 levels were reached. There was a relapse in free T4 levels in 25% of the cases. Another study evaluated the effect of iodide in 21 patients with hyperthyroidism [26]. Thyroid hormone levels started to rise 3 weeks after the start of treatment, but for some others, a euthyroid clinical and biological state was maintained even after 6 weeks. Tan et al. [27] evaluated T4 and T3 serum levels before then 5, 10, and 20 days after treatment with Lugol’s solution in 10 patients with thyrotoxicosis. There was a significant decrease in free T4 at day 5 and day 10, followed by an increase on Day 20, 10 days after the discontinuation of the medication. Lugol has been described in many studies as the optimal preoperative treatment before thyroidectomy for Graves’ disease or for toxic multinodule disease, mainly because of its role in the reduction of thyroid blood flow and intraoperative blood loss along with short‐term control of thyroid function preoperatively, especially in nontolerance or resistance to ATDs [28–31]. Its use during pregnancy is generally restricted to specific, short‐term emergency or preoperative management [4]. Although short‐term iodine administration can effectively control severe thyrotoxicosis, potential risks include the escape phenomenon, rebound hyperthyroidism after withdrawal, and fetal thyroid dysfunction. Mothers may experience gastrointestinal upset, a metallic taste, or allergic reactions [20]. The fetal thyroid gland is significantly more sensitive to iodine than the adult gland. Iodine readily crosses the placenta, leading to several risks. The fetal thyroid does not begin to concentrate iodine until the 10–12th week of gestation. However, exposure to high doses of iodine (such as Lugol’s solution) after this period can cause a prolonged blockade of fetal thyroid hormone production, unlike in adults, who typically escape from this effect within a few days [4]. Additionally, high iodine exposure can cause fetal goiter, which can lead to airway obstruction [4]. Because of the fetal risks, Iodine therapy is reserved for the rapid stabilization of severe maternal thyrotoxicosis when other treatments have failed or are contraindicated. In our patient, these risks were carefully mitigated by limiting treatment to a short duration and ensuring close clinical and biochemical monitoring of both maternal thyroid function and fetal well‐being.

There is no uniform consensus in the guidelines of professional societies on how to adjust the dose of Lugol’s iodine during the treatment of TGT or on the optimal duration of therapy for this indication. The ATA [3] and the European Thyroid Association [20] guidelines similarly recommend Lugol’s solution for short‐term use in hyperthyroid patients, particularly for preoperative preparation before thyroidectomy and for management of thyroid storm. For preoperative preparation in patients with Graves’ disease, Lugol’s 5% solution is administered to reduce thyroid vascularity and intraoperative blood loss, typically at a dose of 5–7 drops (≈0.25–0.35 mL, each drop ≈ 8 mg iodide) three times daily for 10–14 days before surgery. In thyroid storm, it is used to rapidly inhibit thyroid hormone release, generally 5 drops every 6 h, and after administration of ATDs to prevent paradoxical hormone synthesis [3, 20].

**Figure 2:**
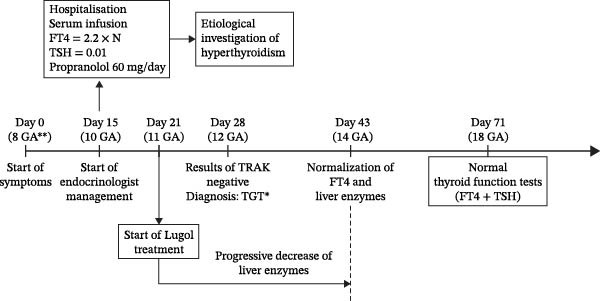
Chronological clinical course and management of transient gestational thyrotoxicosis.  ^∗^TGT: transient gestational thyrotoxicosis.  ^∗∗^GA: gestational age.

To the best of our knowledge, no study has described the use of Lugol in TGT. One study by Jamieson et al. [32] reported the case of a 24‐year‐old pregnant woman diagnosed with Graves’ disease during pregnancy. She was treated with carbimazole, after which she developed joint pain and a cutaneous rash. She was then switched to propylthiouracile (PTU) at a dose of 100 mg/day, after which she developed urticaria, wheezing and was admitted to the hospital for respiratory distress. All medications were interrupted, and she was switched to Lugol’s iodine at 0.3 mL/day. After 4 weeks of treatment, hyperthyroidism symptoms subsided, and a euthyroid clinical and biological state was maintained. This case report proves the efficacy of Lugol’s iodine in treating hyperthyroidism in pregnancy in the case of Graves’ disease. As for our patient, there was a significant improvement in thyroid function after Lugol treatment. However, unlike Graves’ disease, TGT is characterized by a spontaneous improvement by 18 weeks of gestation [9, 33]. Therefore, we cannot completely attribute the role of Lugol to the evolution of our patient’s case. By the end of the first trimester, her symptoms might as well have disappeared spontaneously with the disappearance of TGT that characterizes the second and third trimester of pregnancy. However, she was at 12 weeks of gestation when she reached an euthyroid state but could have had persistent symptoms between 12 and 18 weeks of gestation if Lugol’s iodine has not been started (Figure 2). In the afore‐mentioned case report by Pornchai et al. [17], the patient with TGT associated with severe hepatic cytolysis did not receive antithyroid therapy, and she was completely asymptomatic by the end of the first trimester after simply receiving supportive care. In our patient’s case, hyperthyroidism was complicated with severe hepatic cytolysis, acute pancreatitis, tachycardia, incoercible vomiting, and dehydration. The need for a rapid‐acting treatment such as Lugol was therefore justified. All these findings conclude that Lugol’s iodine can be an interesting option for transient hyperthyroidism, and the best example was that of our patient, having TGT and needing urgent treatment that was compatible with pregnancy. Lugol’s iodine 5% was used because this preparation is routinely compounded in the hospital pharmacy and represents the standard formulation available in our institution. Given the acute clinical presentation and the need for rapid stabilization, treatment choice was guided by clinical judgment rather than a predefined protocol. Further studies are required to determine the most appropriate dosing strategies, particularly in complicated cases where conventional therapies are contraindicated.

## 4. Conclusion

TGT is usually a mild and self‐limited form of hyperthyroidism occurring during the first trimester of pregnancy. Nevertheless, severe presentations may occur, with marked clinical symptoms and significant hepatic biochemical abnormalities requiring urgent management. In the present case, Lugol’s iodine was associated with a rapid decrease in thyroid hormone levels and clinical improvement, alongside normalization of severe hepatic cytolysis and acute pancreatitis, in a context where conventional antithyroid therapies were contraindicated. Therefore, causal inference should be made with caution, and further studies are needed to better define optimal management strategies in cases of complicated TGT.

## Funding

This research did not receive any specific grant from funding agencies in the public, commercial, or not‐for‐profit sectors.

## Consent

Written informed consent for publication of this case report was obtained from the patient.

## Conflicts of Interest

The authors declare no conflicts of interest.

## Data Availability

Data are available upon request from the authors.

## References

[1] Nygaard B. , Hyperthyroidism in Pregnancy, BMJ Clinical Evidence. (2015) 0611. PMC4303927 25614153

[2] Cooper D. S. and Laurberg P. , Hyperthyroidism in Pregnancy, The Lancet Diabetes and Endocrinology. (2013) 1, no. 3, 238–249, 10.1016/S2213-8587(13)70086-X. 24622372

[3] Ross D. S. , Burch H. B. , and Cooper D. S. , et al.American Thyroid Association Guidelines for Diagnosis and Management of Hyperthyroidism and Other Causes of Thyrotoxicosis, Thyroid. (2016) 26, no. 10, 1343–1421. 27521067 10.1089/thy.2016.0229

[4] Alexander E. K. , Pearce E. N. , and Brent G. A. , et al.Guidelines of the American Thyroid Association for the Diagnosis and Management of Thyroid Disease During Pregnancy and the Postpartum, Thyroid. (2017) 27, no. 3, 315–389. 28056690 10.1089/thy.2016.0457

[5] Riley D. S. , Barber M. S. , and Kienle G. S. , et al.CARE Guidelines for Case Reports: Explanation and Elaboration Document, Journal of Clinical Epidemiology. (2017) 89, 218–235, 10.1016/j.jclinepi.2017.04.026. 28529185

[6] Chan L. , Gestational Transient Thyrotoxicosis, The American Journal of Emergency Medicine. (2003) 21, no. 6, 10.1016/S0735-6757(03)00173-6, 506. 14574663

[7] Gridelet V. , Perrier d’Hauterive S. , Polese B. , Foidart J.-M. , Nisolle M. , and Geenen V. , Human Chorionic Gonadotrophin: New Pleiotropic Functions for an « Old » Hormone During Pregnancy, Frontiers in Immunology. (2020) 11, 10.3389/fimmu.2020.00343, 343. 32231662 PMC7083149

[8] Sun S. , Qiu X. , and Zhou J. , Clinical Analysis of 65 Cases of Hyperemesis Gravidarum With Gestational Transient Thyrotoxicosis, Journal of Obstetrics and Gynaecology Research. (2014) 40, no. 6, 1567–1572, 10.1111/jog.12372. 24888917

[9] Goodwin T. M. , Montoro M. , Mestman J. H. , Pekary A. E. , and Hershman J. M. , The Role of Chorionic Gonadotropin in Transient Hyperthyroidism of Hyperemesis Gravidarum, The Journal of Clinical Endocrinology and Metabolism. (1992) 75, no. 5, 1333–1337, 10.1210/jcem.75.5.1430095. 1430095

[10] Glinoer D. , Thyroid Hyperfunction During Pregnancy, Thyroid. (1998) 8, no. 9, 859–864, 10.1089/thy.1998.8.859. 9777758

[11] Burrow G. N. , Thyroid Function and Hyperfunction During Gestation, Endocrine Reviews. (1993) 14, no. 2, 194–202, 10.1210/edrv-14-2-194. 8325252

[12] Lee W.-L. , Yang S.-T. , and Wang P.-H. , Hyperemesis Gravidarum in Pregnancy and Gestational Transient Hyperthyroidism, Taiwanese Journal of Obstetrics and Gynecology. (2023) 62, no. 4, 492–494, 10.1016/j.tjog.2023.04.002. 37407181

[13] Yeo C. P. , Khoo D. H. C. , Eng P. H. K. , Tan H. K. , Yo S. L. , and Jacob E. , Prevalence of Gestational Thyrotoxicosis in Asian Women Evaluated in the 8th to 14th Weeks of Pregnancy: Correlations With Total and Free Beta Human Chorionic Gonadotrophin, Clinical Endocrinology. (2001) 55, no. 3, 391–398, 10.1046/j.1365-2265.2001.01353.x. 11589683

[14] Jansen H. I. , van Herwaarden A. E. , and Huijgen H. J. , et al.Pregnancy Disrupts the Accuracy of Automated fT4 Immunoassays, European Thyroid Journal. (2022) 11, no. 6, e220145. 36219545 10.1530/ETJ-22-0145 PMC9641786

[15] Macle L. , Varlet M. N. , and Cathébras P. , Hyperemesis Gravidarum: A Rare but Potentially Severe Complication of the First Trimester of Pregnancy, La Revue du praticien. (2010) 60, no. 6, 759–764. 20623888

[16] Scappaticcio L. , Longo M. , and Maiorino M. I. , et al.Abnormal Liver Blood Tests in Patients With Hyperthyroidism: Systematic Review and Meta-Analysis, Thyroid. (2021) 31, no. 6, 884–894. 33327837 10.1089/thy.2020.0715

[17] Pornchai A. , Kamalaporn P. , and Sriphrapradang C. , Jaundice Caused by Hyperemesis Gravidarum, Ochsner Journal. (2022) 22, no. 4, 372–378, 10.31486/toj.22.0019. 36561106 PMC9753955

[18] Fu Q. , Cui J. , Zhang Y. , Liu J. , and Zhang X. , Case Report: A Case of Acute Pancreatitis With Myocarditis and Thyrotoxicosis, Frontiers in Cardiovascular Medicine. (2025) 12, 10.3389/fcvm.2025.1527970, 1527970. 40662134 PMC12256499

[19] Malagola E. , Chen R. , and Bombardo M. , et al.Local Hyperthyroidism Promotes Pancreatic Acinar Cell Proliferation During Acute Pancreatitis, The Journal of Pathology. (2019) 248, no. 2, 217–229, 10.1002/path.5247. 30714146

[20] Kahaly G. J. , Bartalena L. , Hegedüs L. , Leenhardt L. , Poppe K. , and Pearce S. H. , European Thyroid Association Guideline for the Management of Graves, European Thyroid Journal. (2018) 7, no. 4, 167–186, 10.1159/000490384. 30283735 PMC6140607

[21] Pearce E. N. , Management of Thyrotoxicosis: Preconception, Pregnancy, and the Postpartum Period, Endocrine Practice. (2019) 25, no. 1, 62–68, 10.4158/EP-2018-0356. 30289300

[22] Markou K. , Georgopoulos N. , Kyriazopoulou V. , and Vagenakis A. G. , Iodine-Induced Hypothyroidism, Thyroid. (2001) 11, no. 5, 501–510, 10.1089/105072501300176462. 11396709

[23] Calissendorff J. and Falhammar H. , Lugol’s Solution and Other Iodide Preparations: Perspectives and Research Directions in Graves’ Disease, Endocrine. (2017) 58, no. 3, 467–473, 10.1007/s12020-017-1461-8. 29075974 PMC5693970

[24] Uchida T. , Goto H. , and Kasai T. , et al.Therapeutic Effectiveness of Potassium Iodine in Drug-Naïve Patients With Graves’ Disease: A Single-Center Experience, Endocrine. (2014) 47, no. 2, 506–511, 10.1007/s12020-014-0171-8. 24493028

[25] Takata K. , Amino N. , and Kubota S. , et al.Benefit of Short-Term Iodide Supplementation to Antithyroid Drug Treatment of Thyrotoxicosis due to Graves’ Disease, Clinical Endocrinology. (2010) 72, no. 6, 845–850, 10.1111/j.1365-2265.2009.03745.x. 19912243

[26] Phillppou G. , Koutras D. A. , Plperlngos G. , Souvatzoglou A. , and Moulopoulos S. D. , The Effect of Iodide on Serum Thyroid Hormone Levels in Normal Persons, in Hyperthyroid Patients, and in Hypothyroid Patients on Thyroxine Replacement, Clinical Endocrinology. (1992) 36, no. 6, 573–578, 10.1111/j.1365-2265.1992.tb02267.x. 1424182

[27] Tan T. T. , Morat P. , Ng M. L. , and Khalid B. A. , Effects of Lugol’s Solution on Thyroid Function in Normals and Patients With Untreated Thyrotoxicosis, Clinical Endocrinology. (1989) 30, no. 6, 645–649, 10.1111/j.1365-2265.1989.tb00270.x. 2591064

[28] Hedberg F. , Cramon P. K. , Bränström R. , Falhammar H. , and Calissendorff J. , Assessing the Impact of Short-Term Lugol’s Solution on Toxic Nodular Thyroid Disease: A Pre-Post-Intervention Study, Frontiers in Endocrinology. (2024) 15, 10.3389/fendo.2024.1420154, 1420154. 39119004 PMC11306062

[29] Tsai C. H. , Yang P. S. , Lee J. J. , Liu T. P. , Kuo C. Y. , and Cheng S. P. , Effects of Preoperative Iodine Administration on Thyroidectomy for Hyperthyroidism: A Systematic Review and Meta-Analysis, Otolaryngology–Head and Neck Surgery. (2019) 160, no. 6, 993–1002, 10.1177/0194599819829052. 30721111

[30] Barranquero A. G. , Muñoz de Nova J. L. , and Gómez-Ramírez J. , et al.Effect of Preoperative Potassium Iodide Administration on Graves’ Disease Surgery: A Propensity Score Analysis, The American Journal of Surgery. (2021) 222, no. 5, 959–963, 10.1016/j.amjsurg.2021.04.023. 33941360

[31] Calissendorff J. and Falhammar H. , Rescue Pre-Operative Treatment With Lugol’s Solution in Uncontrolled Graves’ Disease, Endocrine Connections. (2017) 6, no. 4, 200–205, 10.1530/EC-17-0025. 28325735 PMC5434745

[32] Jamieson A. and Semple C. G. , Successful Treatment of Graves Disease in Pregnancy With Lugol’s Iodine, Scottish Medical Journal. (2000) 45, no. 1, 20–21, 10.1177/003693300004500107. 10765530

[33] Caffrey T. J. , Transient Hyperthyroidism of Hyperemesis Gravidarum: A Sheep in Wolfs Clothing, The Journal of the American Board of Family Medicine. (2000) 13, no. 1, 35–38, 10.3122/jabfm.13.1.35. 10682883

